# A Review of the Preservation of Hard and Semi-Hard Cheeses: Quality and Safety

**DOI:** 10.3390/ijerph18189789

**Published:** 2021-09-17

**Authors:** Ana Isabel Nájera, Sonia Nieto, Luis Javier R. Barron, Marta Albisu

**Affiliations:** 1 Lactiker Research Group, Faculty of Pharmacy, Universidad del País Vasco/Euskal Herriko Unibertsitatea, 01006 Vitoria-Gasteiz, Spain; luisjavier.rbarron@ehu.eus; 2 Efficient and Sustainable Processes Department, Bizkaia Technology Park, AZTI, P.O. Box 609, 48160 Derio, Spain; snieto@azti.es

**Keywords:** ripened cheese, shelf-life, security, storage improvement, conservation

## Abstract

Cheese is a dairy product with potential health benefits. Cheese consumption has increased due to the significant diversity of varieties, versatility of product presentation, and changes in consumers’ lifestyles. Spoilage of hard and semi-hard cheeses can be promoted by their maturation period and/or by their long shelf-life. Therefore, preservation studies play a fundamental role in maintaining and/or increasing their shelf-life, and are of significant importance for the dairy sector. The aim of this review is to discuss the most effective methods to ensure the safety and sensory quality of ripened cheeses. We review traditional methods, such as freezing, and modern and innovative technologies, such as high hydrostatic pressures, chemical and natural vegetable origin preservatives, vacuum and modified atmosphere packaging, edible coatings and films, and other technologies applied at the end of storage and marketing stages, including light pulses and irradiation. For each technology, the main advantages and limitations for industrial application in the dairy sector are discussed. Each type of cheese requires a specific preservation treatment and optimal application conditions to ensure cheese quality and safety during storage. The environmental impact of the preservation technologies and their contribution to the sustainability of the food chain are discussed.

## 1. Introduction

Extending the shelf-life of food products has long been a significant concern for the dairy industry. Traditionally, this utilized natural atmospheric conditions, such as sun drying in summer, and cold and freezing in winter, and the advantages of natural fermentation for cheese preservation [1]. In recent years, active and intelligent packaging and non-thermal technologies have emerged to prevent the deterioration of perishable food products.

Cheeses can be made from different types of milk and processing technologies, and ripened for different periods, resulting in numerous varieties with a wide diversity in terms of texture, flavor, and shape. Hard and semi-hard cheeses are versatile nutrient-dense dairy products. Although these are highly valued, from a health perspective, significant controversy exists among consumers and in the scientific community. Cheese contains saturated fatty acids, cholesterol, and salt, which have been associated with cardiovascular disease (CVD) risk; however, cheeses also contain a range of nutrients that are potentially beneficial to health [2,3,4]. Recent studies indicate that not all saturated fatty acids raise the content of cholesterol in plasma in the same manner, and that some saturated and trans fatty acids in cheese may play a beneficial role in human health. Moreover, other healthy components present in cheeses are conjugated linoleic acids (CLA) and phospholipids from the fat globular membrane [2,3,5]. In this regard, fermented dairy products have been proposed as functional foods with a cholesterol-lowering effect and, therefore, with a protective effect against CVD compared to non-fermented dairy products. The so-called French paradox, in which low mortality from coronary heart disease has been observed despite the high cheese intake by consumers, is an important consideration in the nutritional assessment of cheese consumption [4,6]. A recent study reported that increased dairy consumption may contribute to a lifestyle associated with a reduction in CVD risk [7]. In addition, bioactive components are generated during cheese fermentation, such as gamma aminobutyric acid, which favors the survival of probiotic microorganisms [8].

Cheese also contains digestible proteins of high biological value. During cheese ripening and food digestion, caseins are hydrolyzed and peptides with antioxidant capacity are generated. The addition of an adjunct culture and a long ripening time increases the formation of peptides and enhances the antioxidant capacity. Some of these peptides are also a prominent source for nutraceutical functional foods [2]. Many of the fat-soluble vitamins are held in cheese fat. Although some water-soluble vitamins may be lost during whey drainage, folate, niacin, B12, and riboflavin remain in sufficient quantities in the cheese matrix to have a significant effect on human nutrition. In addition, propionic acid bacteria synthesize considerable levels of vitamin B12 in hard cheeses [2]. Ripened cheeses are an important source of minerals, particularly calcium (Ca) and phosphorus (P). The calcium in cheese is highly bioavailable due to the formation of complexes with peptides and its high content promotes fat excretion and reduces blood pressure. Similarly, cheese is a good source of this mineral for lactose intolerant individuals [6]. The acid phosphatase enzymes aid in the generation of phosphorylated peptides, which also have beneficial health effects. In this regard, IDF [9] indicates that the elimination of dairy products from diets may result in a loss of calcium and other essential nutrients for part of the population. Sodium (Na) is a nutrient that should be reduced in the diet, but it is estimated that cheese adds only about 5–8% of the total Na intake [2]. A recent study reported that the intake of Na from cheese may prevent induced vessel alteration by reducing oxidative stress, rather than the intake of Na from non-dairy foods. Therefore, cheese intake may be an effective dietary strategy to reduce the risk of CVD in healthy older adults without salt-sensitive blood pressure [7]. Therefore, cheese is a highly valued product by consumers and its consumption has increased in recent years because, among other reasons, a significant proportion of consumers perceive it as a healthy food.

The European Union (EU) is the world’s leading cheese producer, followed by the United States (USA). Combined, the two areas account for around 70% of the global production. In total, EU countries produced 8959 million metric tons of cheese in 2010, and in 2020 the EC production was 10,350 million metric tons [10]. Global cheese production is expected to increase progressively until 2027, with developed countries increasing milk production by 9%. Of this increased milk production, 37% is expected to be used to make cheese [11]. Cheese consumption has grown in all global regions; USA and, in particular, the EU, are the main cheese consuming areas. In 2010, around 17 kg of cheese per person/per year was consumed globally, and this amount increased to 18.44 kg cheese per person in 2020 [12]. The EU has recently expanded cheese exports to Canada and the Russian Federation, and it is expected that China and Egypt will double cheese imports by 2027. In addition, other regions, such as the Middle East and North Africa, will become key destinations, accounting for 19% of global cheese imports by 2027, because cheese has been progressively introduced into the diets of their consumers [11]. The increase in global cheese consumption is also due to changes in food habits, particularly in East Asia, where the use of cheese as an ingredient in snacks and processed meals has increased. Thus, cheese preservation methods are highly important for the dairy industry in order to increase cheese consumption.

Cow’s milk cheeses are produced in the greatest quantity throughout the year, whereas the production of small ruminant milk cheeses is lower and seasonal [13,14,15]. Evidently, this seasonality affects the regularity of cheese manufacture during the year, in addition to the milk composition. In this respect, because the manufacturing time is not long, the shelf-life of these cheeses during which they retain their optimal sensory characteristics is short [16,17]. Therefore, an adequate preservation method is extremely important for hard and semi-hard cheeses, to increase the availability for a longer period without changing their sensory characteristics.

The *Codex Alimentarius* classifies cheese varieties according to their composition and consistency parameters and ripening times, taking into account the percentage of moisture without fat. The extra-hard specification refers to cheeses with a moisture content less than 51%. Cheeses with a moisture content between 49–56% and 54–69% are called hard and semi-hard cheeses, respectively. Soft cheeses have a moisture content higher than 67% [18]. The Spanish regulation indicates that semi-hard (semi-cured) cheeses must have a minimum ripening time of 20 days (cheese weight less than 1.5 kg) or 35 days (cheese weight greater than 1.5 kg), depending on the cheese weight when the cheeses are marketed. By comparison, hard (cured) cheeses must be ripened for at least 45 or 105 days, depending on whether the cheese weighs less than 1.5 kg [19].

Changes in protein and fat content in cheese during ripening are responsible for many important nutritional and sensory properties. Chemical reactions such as lipolysis may also occur during cheese storage. This is an important biochemical reaction responsible for generating the desired flavor of many cheese varieties. However, excessive amounts of short-chain fatty acids can lead to a rancid off-flavor in ripened cheeses [20]. In contrast, proteolysis breaks down proteins into peptides and amino acids, generally improving cheese texture and flavor. The hydrolysis of peptides and catabolism of amino acids, fatty acids, and lactic acid results in the formation of volatile compounds that strongly influences the cheese flavor. However, this hydrolytic process can also lead to an increase in the concentration of substances that are toxic to human health, such as biogenic amines [21]. Cheese ripening followed by a long storage period may cause economic losses to cheese makers if degradation processes occur due to inadequate storage conditions [22]. Therefore, it is highly important to avoid the deterioration of the dairy product at all stages. Although hard and semi-hard cheeses have a reasonably long shelf-life, this may be limited by several factors during the maturation and storage periods, so effective conservation techniques should be employed prior to commercialization [23]. Microbial lipases and proteases can generate off-flavors, strange colorings, and mycotoxins, which decrease the cheese quality and safety [17]. Moreover, the cost of preservation treatments to prevent or control the surface growth of molds and yeasts in cheeses is high [24]. These treatments aim to reduce spoilage microorganisms and eliminate pathogens without affecting the lactic bacteria responsible for the final characteristics of the cheese. Exposure of cheese to heat, oxygen, and light stimulates enzymatic oxidation reactions that can produce different degradation processes, such as discoloration, production of off-flavors, nutrient loss, and formation of toxic substances [23]. Light-induced oxidation of photosensitive substances in cheese, such as riboflavin and carotenoids, also requires the presence of oxygen [25,26]. The removal of oxygen in the atmosphere surrounding the cheese surface may prevent degradation processes during storage and marketing. The characteristics of whole cheeses are also susceptible to changes due to environmental factors, when sold unpackaged. Therefore, optional preservation methods to be applied during storage and marketing should be specifically investigated for each cheese variety.

Due to these reasons, cheese preservation plays a fundamental role in increasing the shelf-life of the product, and is of significant importance to the cheese industry. The aim of this review is to discuss the main contributions presented in the scientific literature on the methods to preserve hard and semi-hard cheeses, from the traditional to the most innovative. For the different technologies, in addition to reviewing the information reported about the impact of the preservation methods on the cheese quality and safety, the main benefits and limitations for their industrial application is also discussed. Other aspects of interest that are taken into account in this review are the environmental impact, the contribution to the sustainability of the food chain, and the consumer preferences.

The bibliographic search was performed in the following electronic databases: Web of Science (Clarivate Analytics, London, UK), Scopus (Elsevier, Amsterdam, The Netherlands), and Google Scholar (Google, Mountain View, CA, USA).

The search terms used were the following: [Cheese OR Hard cheese OR Semi-hard cheese OR Ripened cheese OR Ewe cheese OR Goat cheese] AND [Composition OR Nutrition OR Production OR Consumption OR Freezing OR High hydrostatic pressure OR Additives OR Natural preservatives OR Vacuum packaging OR Modified atmosphere packaging OR Edible coating OR Active packaging OR Light-emitting diode OR Pulsed light OR Irradiation].

The search strategy focused on the following aspects of interest: preservation technology, study design, conditions, type of cheese, shelf-life, changes produced, main results, benefits, and limitations.

In the first step, the studies were selected based on the title and abstract. In the second stage, the full text of the articles was read. Backward citation tracking was used to find other relevant studies. The search was last updated on June 2021. No time or language filters were applied. Exclusion criteria included irrelevance and studies referring to other different types of cheeses (no hard or semi-hard cheeses).

## 2. Freezing and Frozen Storage

Freezing allows food to be preserved for long periods, while also maintaining a high nutritional quality. This process consists of lowering the food temperature below its freezing point, which causes water crystallization, significantly inhibiting microbial growth and biochemical reactions. The formation of ice crystals can cause physical damage to the food structure [27]. This technique is widely used in the food industry, and authors have described the influence of freezing and frozen storage on the characteristics of hard and semi-hard cheeses [14,28].

This storage method may enable the accumulation of a long-term stock cheese reserve for the dairy industry, and this advantage is more important for seasonal production cheeses, such as ewe’s and goat’s milk cheeses [13,14,15]. Thus, to regulate the market for these seasonal cheeses, several frozen storage tests have been carried out, with little success. Freezing milk or freezing concentrated milk before cheesemaking have been described in the literature, but significant organoleptic defects have been observed in cheeses when freezing at −15 and −27 °C. Total viable counts and coliforms decline faster at −15 °C than at −27 °C [29]. However, some authors [30] reported that good quality cheese can be obtained from frozen sheep milk at −15 and −25 °C for up to 6 months, without influencing cheese yield or composition. Freezing the cheese curd has traditionally been considered as a useful option to regulate the seasonal cheese market. In this regard, it was found that freezing produced significant changes in the microstructure of Crottin de Chavignol goat cheese and reduced the total lactic acid bacteria (LAB) count by 2 log units [31]. Hispánico cheese of satisfactory texture and sensory properties was obtained by mixing frozen ewes’ milk curd with fresh cows’ milk curd with no significant differences in LAB count [32]. However, few authors have studied the impact of the freezing process on the characteristics of hard and semi-hard cheeses [14,28]. Freezing of ripened cheeses has also been attempted to slow the over-ripening process and extend the cheese shelf-life by inhibiting or reducing enzyme activities and chemical reactions [16]. Results reported for Motal cheese showed that the storage at −18 °C reduced the formation of excessive amounts of free fatty acids (FFAs) and led to volatile compounds and little decline in LAB count, resulting in the maintenance of the product quality and extended shelf-life [28].

The optimization of the freezing method, storage conditions (temperature and time), and thawing are crucial factors, because the preservation should preserve the desirable qualities of the final product. In addition, the product quality depends on the type of cheese, its composition, and ripening time [33,34]. Depending on the transition from water to ice, the internal structure of the cheese matrix can be damaged, thus altering cheese texture properties [35]. Fat, moisture, and salt contents play a critical role in frozen cheese properties. Thus, low moisture cheeses resist frozen storage better than high moisture cheeses. In addition, a higher fat content helps cheese to resist structural changes during frozen storage. A high fat content in cheese was reported to maintain the ice crystal size below 50 µm in diameter [36]. By comparison, low protein hydration during the freezing process is one of the main causes of defects in the texture, resulting in insufficient elasticity, and a crumbly and powdery product. It was reported that, at −20 °C, the water-holding capacity of proteins and the hydrophilic properties of curds were preserved in some Russian semi-hard cheese varieties. When using lower freezing temperatures, a transition of the micelle-bound water into ice occurred, and there were structural changes that led to the appearance of an additionally elastic and crumbly cheese consistency [34].

Fontecha et al. [37] studied the textural and microstructural characteristics of semi-hard sheep cheeses submitted to slow freezing at −35 °C (plate freezer, 1.55 cm/h) and fast freezing at −80 °C (liquid nitrogen vapor, 4.0 cm/h), and subsequent ripening after thawing. In both treatments, cheeses were frozen up to −20 °C and storage at −20 °C for four months. The results showed that a slow freezing rate together with a longer period of frozen storage increased the deformation of the cheese matrix and decreased the share strength and firmness of the thawed cheeses. Slowly frozen cheeses presented a more extensive breakdown in their microstructure with longer cracks than fast frozen cheeses, for which textural properties were closer to those of the unfrozen cheeses. Nevertheless, the subsequent ripening process tended to offset the changes in the cheese matrix and to equalize the characteristics of the final products, both frozen and non-frozen. Tejada et al. [13,38] investigated the effect of the freezing rate (slow at −20 °C and fast at −82 °C) and frozen storage time (9 months at −20 °C) on the properties of a ewe’s milk cheese after 90 ripening days. No significant effect was observed for the two freezing treatments on the chemical and microbiological characteristics of the cheeses, and graininess of the cheese was only slightly greater in slowly frozen cheeses. There were no significant differences compared with control cheeses and between the two freezing rates for total viable counts and enterococci, *Enterobacteriaceae*, coliforms, staphylococci, molds/yeasts, and micrococci. Leuconostoc and lactobacilli showed a gradual decrease that was more accentuated at the end of the storage period (9 months). This study concluded that chemical and microbiological composition, and sensory properties of the cheeses, did not change after six months storage at −20 °C. Similar results were published for a 180 day ripened Manchego-type cheese stored at −20 °C for six months. The microbiological results exhibited similar counts for total viable microorganisms, LAB, and molds and yeasts, in contrast to micrococci and staphylococci that decreased during the frozen storage [14].

Similarity, freezing at −20 and −30 °C after 42 ripening days and frozen storage at −10 and −20 °C did not affect the content of moisture, fat, and total nitrogen in semi-hard Serpa sheep cheeses. However, higher values of non-protein nitrogen and hardness were found, and some color parameters changed (more luminous and more yellow-green), in the frozen cheeses after 12 months of storage. In addition, the damage reflected in cheese properties was diminished using storage temperatures of −20 °C in comparison to −10 °C. In this case, slow or fast freezing did not affect the physico-chemical cheese properties [39].

The usefulness of the freezing process for the conservation of some goat’s milk cheeses with different ripening times has also been reported [40,41]. Minimal flavor effects were observed in a goat cheese variety after six months storage at −20 °C. Five years of frozen storage at −20 °C had minimal effects on cheese flavor, and only a more granular and pasty texture was observed [15]. The protein bound water does not crystallize at −20 °C, and its physical properties remain unchanged [34]. Thus, this temperature suits the maximum level of maintenance of the protein structure, whereas other freezing treatments result in low quality cheeses due to the chemical reactions caused by the presence of a high quantity of the unfrozen solution and by the freezing of the bound water.

The low-temperature processing and storage ensure longer cheese preservation of up to a year or more, and can be beneficial for the profitability of the dairy sector and beneficial for the environment (lower energy consumption) [34]. Additionally, it was described that storage at freezing temperatures of Motal semi-hard cheese hindered the formation of biogenic amines after 180 days of storage, which contributed to healthier aged cheeses [28]. However, this conservation technique can have several drawbacks. To avoid freezing burns, cheeses must be packaged, and this pre-treatment involves the use of waterproof materials such as plastics, which is not a preferred option due to environmental and sustainability concerns, in addition to current legislative restrictions. Furthermore, the cold chain may break down during storage or transport and cause alterations in the cheese.

In general, the flavor and nutritional characteristics of the cheeses are not altered during frozen storage. In order to preserve cheese texture, several studies propose the use of a storage temperature of −20 °C rather than lower temperatures. Although freezing of milk, curd, or cheese has been proposed as an interesting option to regulate the market of seasonal products, this preservation technology is not currently used industrially for ripened cheeses.

Different innovative freezing processes are currently being tested to improve the quality of frozen foods. Johnston [42] investigated the potential of pressure-shift freezing at −20 °C at 200 MPa followed by pressure thawing of the cheese, with the aim of maintaining, as much as possible, the rheological characteristics of some cheese varieties. Although this innovative treatment can partially counteract changes in the rheological properties of Cheddar cheese, the frozen cheeses were still distinguishable from fresh cheeses in terms of texture parameters, such as deformation and compression at fracture. Many of these particular freezing techniques are still in the industrial development phase, and involve a high capital cost. For this reason, it is important to consider the product quality versus cost for the application of these preservative techniques to the dairy industry [27].

## 3. High Hydrostatic Pressure (HHP) Processing

HHP processing is probably the most advanced non-thermal emerging technology used for food processing at present time. Equipment for large-scale production with HHP is now commercially available, demonstrating the fast development that is taking place in the food industry sector [43]. During HHP treatment, the product is subjected for a short time period (10–20 min) to a very high pressure level (400–600 MPa is normally used at the industrial scale) and a temperature below 45 °C. Based on the isostatic principle, pressure applied in HHP treatments is transmitted instantaneously and uniformly throughout food, regardless of size, shape, and composition [44]. This conservation treatment extends food shelf-life, and preserves nutritional characteristics and sensory attributes [45,46].

HHP treatments have been described as being effective in reducing pathogenic and spoilage microorganisms in cheeses, and can produce biochemical changes due to alteration of proteolysis and lipolysis activities. Therefore, the shelf-life varies because ripening can be accelerated using HHP treatments with low to moderate pressures (200–400 MPa), and storage cost can also be reduced [47,48]. In addition, it has been reported that pressures higher than 500 MPa cause a proteolysis reduction that prevents over-ripening of fresh, soft, and semi-hard cheeses, and slows chemical and enzymatic reactions that continue during refrigerated storage, at retail locations and at home [49,50,51].

Inactivation of microorganisms is due to morphological, biochemical, and genetic alterations that take place under high pressures. Gram positive (+) bacteria are more resistant to high pressure than Gram negative (−) bacteria, because the former are inactivated with treatments of 500–600 MPa, whereas 300–400 MPa are needed for the latter, in both cases using 10 min and 25 °C. Therefore, LAB present in milk can survive, whereas pathogenic microorganisms are eliminated. Rod-shaped bacteria are more sensitive than cocci, and endospores are highly resistant to HHP treatments, particularly *Clostridium* spp., which usually requires pressures around 1000 MPa. This is a significant issue when aiming to prevent late blowing defects in ripened cheeses. Because yeast and mold vegetative forms are the most pressure sensitive [52], HHP can prevent their growth during storage [53].

Microorganisms are more sensitive to high pressure treatments in a buffer solution than in the cheese matrix. A starter with *Lactococcus lactis* in a buffer solution was subjected to HHP in the range of 100–400 MPa for 20 min to produce microbial lysis and release enzymes to accelerate cheese maturation. Simultaneously, 1-day-old cheese with the same untreated strains was submitted to HHP. In the latter case, the HHP conditions did not improve starter autolysis [54]. A HHP treatment of 200 MPa for 20 min was applied either to starters (*Streptococcus thermophylus*, *L. lactis*, and *Lactobacillus bulgaricus*) or to ripened sheep cheese at the beginning of ripening, and cheese characteristics were compared with those of untreated control cheeses. All cheese samples were stored for 90 days at 4 °C. Cheeses from HHP-treated starters presented the higher sensory scores, and no bitterness was detected during storage. Secondary proteolysis was higher in these cheeses than in the other cheese samples, whereas the HHP-treated cheeses showed the highest aminopeptidase activity [47].

When high pressure treatment is applied to milk, microbiological quality comparable to that of pasteurized milk can be achieved [55]. Thus, thermal milk pasteurization can potentially be replaced by HHP treatment for cheesemaking [56]. However, milk HHP treatments can modify the physico-chemical structure of proteins, causing the fragmentation of casein micelles and denaturation of whey proteins, mainly due to the generation of non-covalent disruptions [57,58,59].

HHP processing is applied to cheese rather than to starters or milk [60]. HPP treatment has been shown to be an effective tool in eliminating cheese-borne pathogens. Such is the case with *Escherichia coli* tested in model semi-hard cheeses [61] or *Listeria* spp. in Ibores cheese [62]. However, the optimal processing parameters and the best time for HPP application may vary depending on cheese variety [51]. In this regard, cheese defects of microbial origin can be controlled by HPP treatments. Coliform growths responsible for the early blowing defect, particularly in cheese made from raw milk, can be limited using moderate HPP treatments (200–400 MPa) [45,50,63]. It was reported that pre-treatment of cheese at moderate pressure (300–500 MPa) induced the germination of *Clostridium tyrobutyricum* spores but a further high-pressure HPP treatment increased the microbial lethality [64]. In vacuum-packed 7-day ripened sheep cheese, the HHP treatment at 300 MPa avoided the late blowing defect, but these pressurized cheeses showed a fracturable texture and low color, and the generation of certain volatile compounds was retarded [48].

Moschopoulou et al. [60] indicated that HHP treatment at 200 or 500 MPa applied to sheep cheese after 15 ripening days did not modify the cheese chemical composition. HHP treatments at 200 MPa were sufficient to inhibit coliform growth, and 500 MPa significantly delayed the growth of other microorganisms (total aerobic mesophiles, thermophilic starters, and non-starter bacteria (NSLAB). Arqués et al. [63] found a significant reduction of spoilage microorganism using HHP treatments (300–400 MPa for 10 min) applied to raw milk ewe’s cheeses after 2 and 50 ripening days. HHP applied to 50 day matured cheeses did not affect their sensory properties, whereas the treatment applied at early ripening stages had a negative effect on cheese flavor. Similar results were obtained when pressures of 400 and 600 MPa were applied at three different ripening times (1, 30, or 50 ripening days) to raw goat’s milk cheeses. Both HHP treatments reduced undesirable microorganisms in all cases. However, both HHP treatments applied at the first ripening day changed the texture profile, appearance, and flavor. In the case of a 600 MPa treatment applied to cheese at 30 and 50 ripening days, no sensory and proteolytic changes were observed, whereas spoilage microorganisms experienced a greater reduction. The content of short chain FFAs only decreased in cheeses treated at 600 MPa at the first ripening day. Medium and long FFA content did not vary with any of the HHP treatments [45,65]. Inácio et al. [56] reported a significant reduction in the microbial count of *Enterobacteriaceae*, *Listeria innocua*, molds and yeasts in raw milk ewe’s cheeses treated with high pressure. HHP treatment (400–600 MPa) applied to cheese at 45 ripening days did not significantly modify its physico-chemical characteristics, although lipid oxidation was reduced in comparison with non-pressurized cheeses after 100 ripening days. Treatments of 400 MPa for 20 min in Cheddar cheese slurries inoculated with microorganisms produced a 3 log unit reduction in *Staphylococcus aureus*, and 6 and 7 log reductions in *E. coli* and *Penicillium roqueforti*, respectively, in addition to a reduction in the growth of molds and yeasts [53].

The application of HPP to cheese during ripening may lead to either the acceleration or the reduction of the ripening process. HHP treatments can influence cheese proteolysis by modifying the conformational structure of the proteins, activation or inactivation of proteinases, and inhibition or acceleration of the microbial growth and metabolism [66]. Studies showed that pressure intensity and the time of application are crucial to maintain the cheese’s texture and flavor characteristics [54,60,65]. Changes during ripening can be due to an increase in primary or secondary proteolysis when using 200–400 MPa HHP treatments. The mechanism by which ripening is accelerated is still unclear and further research is needed. HHP treatments induce conformational changes in proteins, affecting the enzyme modulation sites [47]. In this regard, some authors have attributed the enhanced proteolysis to cell lysis and enzyme release. In addition, a higher pH value (0.1–0.2 units) in HPP-treated cheese is more favorable for starter peptidase activity and can improve proteolysis during cheese ripening [67,68]. Proteinases and peptidases responsible for peptides and free amino acid (FAA) release can be modified by HHP treatments and, consequently a reduction in ripening time can be expected in most cases [47,48,69].

Edam cheese proteolysis was examined with the aim to determine the possibilities of accelerating the cheese ripening process, or cheese preservation. Cheese samples were subjected to pressures of 200 and 400 MPa, after salting and after four, six and eight ripening weeks. Control samples were traditionally ripened Edam cheeses. Pressures of 200 and 400 MPa had no significant effect on proteolysis, although HHP treatments improved cheese consistency [70]. By comparison, a 100-fold reduction in LAB growth together with a retarded growth of NSLAB was observed in a 180-day-old Cheddar cheese when 400 MPa HHP treatment was applied for 10 min on the first day post-processing. In this case, there was little effect on the primary proteolysis, because the activities of chymosin and plasmin were not affected by the treatment. The HHP-treated cheeses showed color alteration. After 90 ripening days, these cheeses presented higher scores in some sensory attributes (animal cooked fat flavor and butter odor), but the overall flavor intensity was lower in HHP-treated cheeses than in untreated cheeses [71]. Several references describe the effect of HHP on different types of cheese during ripening; Cheddar [54,72], Hispánico [73,74,75,76], Serena [77], ewe’s milk [68], and Reggianito cheese [69]. Moreover, the effect of HHP (600 MPa) on partial or total inactivation of microorganisms and enzymes is effective in retarding proteolysis and lipolysis, and in reducing the formation of some undesirable volatile compounds [51].

The reduction in biogenic amine content induced by HPP is mainly due to the elimination of NSLAB with amino acid decarboxylation activity [51]. HHP treatments of 400 and 600 MPa applied for 5 min to ewe’s milk cheeses at 21 or 35 ripening days were found to be useful to reduce the formation of biogenic amines in 60-day-old cold-stored cheeses. The 600 MPa level was more effective than that of 400 MPa. The decline in biogenic amines was attributed to reduced counts of enterococci and lactobacilli in HHP-treated cheeses, the decrease in decarboxylase activity, and the low concentration of FAAs [78]. From day 180 onwards, similar HHP effects on the biogenic amine content were found in raw cow’s milk cheese, with similar HHP treatment applied at 14 or 21 ripening days. Lower short chain FFA concentrations in cheeses treated with 400 or 600 MPa were found in comparison to untreated cheeses after 140 ripening days; this may be due to a lower esterase activity in HHP-treated cheeses. By comparison, no significant differences were observed in flavor preference and intensity between HHP-treated and untreated cheeses, but bitterness was higher from day 60 onwards in 400 MPa-treated cheeses [50,79].

To achieve more regular cheese production throughout the year and reduce the storage time, particularly for ewe’s and goat’s cheeses, HHP has been applied to raw milk curd followed by frozen storage. The ewe’s raw milk curd was treated at 400 or 500 MPa, and goat’s raw milk curd at 400 MPa, for 10 min and kept frozen at −24 °C up to five months. Cheese manufacture was a mix of 20% (by weight) HHP-treated ewe’s curd and 80% of freshly made curd from pasteurized cow’s milk. In goat cheesemaking, the mixture of HHP-treated and freshly curd was 30:70. Control cheeses were made with the same curd mixtures but without HHP treatment. For both sheep and goat cheeses, at day 60, no differences were found between control and experimental cheeses in total viable microbial counts, Gram (−) bacteria and LAB growth, but staphylococci presented higher counts in 400 MPa-treated ewe’s cheeses than in the other cheeses. Aminopeptidase activity showed the same levels in pressurized cheeses as in control cheeses for both sheep and goat cheeses. Proteolysis was higher in cheeses made with all pressurized curds, and a greater release of FAAs in ewe’s cheeses treated at 500 MPa was observed. Esterase activity and total FFAs showed higher levels in treated cheeses at 400 MPa than in control cheeses. Long chain-FFAs were 11% lower in goat-pressurized cheese, whereas no significant differences were found for short- and medium-chain FFAs. In the same manner, no differences were observed in the sensory attributes between control and pressurized cheeses, with the exception of flavor quality scores, which were higher in goat-pressurized curd cheeses. In addition, these authors indicated a potential benefit for the cheese industry by increasing the yield and reducing the ripening time of the pressurized ripened cheeses [80,81].

The results described above indicate that HHP treatments have been applied to starters, milk, curds, and hard and semi-hard cheeses at different ripening days. HHP treatment at moderate doses (200–400 MPa) can be a reliable technique to reduce or eliminate cheese pathogens and undesirable microorganisms that cause defects in cheeses. However, spore-forming bacteria, such as *Clostridium* spp., need higher pressures (over 1000 MPa) to be effective. Many studies on HHP treatments have focused on accelerating or delaying cheese ripening in order to diminish storage costs or produce cheeses with optimal sensory characteristics after long storage periods. Moderate pressures (100–400 MPa) tend to accelerate proteolysis and, in consequence, shorten ripening and storage time, so these treatments could be especially useful for hard cheeses. Higher pressures (>500 MPa) are usually more effective in delaying proteolysis and lipolysis, and may be useful in the case of semi-hard cheese production. Ripening modulation can benefit small ruminant seasonal cheeses, and may be used to overcome seasonal shortages or production surpluses. Furthermore, the optimal ripening time for applying HHP treatment is another factor that should be taken into account. In this regard, the pressurization applied during the first ripening days leads to significant biochemical and sensory changes in cheeses. When the pressurization treatment is applied at later ripening stages, cheese flavor is little affected. The color, flavor, and texture of pressurized cheeses are often the most affected sensory parameters, independently of the HHP treatment and application ripening time.

A limiting factor is the high equipment cost. Thus, a significant proportion of newly installed HPP equipment operates under a toll service regime [51,52,82]. From an environmental perspective, another unfavorable aspect is the use of plastic packaging materials necessary to apply the pressurization treatment. HHP has good potential to be applied to ripened cheeses in order to prolong their shelf-life, but further studies are necessary for each cheese variety to optimize HHP conditions, and to verify the effects of pressurization treatments on biochemical, textural, and sensory characteristics of cheeses.

## 4. Food Additives

The direct addition of additives to foods is one of the simplest and oldest preservation techniques used to extend their shelf-life. At present, cheese preservation is often undertaken via chemical or biological additives. These substances are added to cheese in order to avoid defects caused by microorganisms, and extend the cheese’s shelf-life, improve its physical properties and chemical composition, and preserve its nutritional value [23,43].

### 4.1. Regulated Additives in the European Union for Ripened Cheeses

Food preservatives approved in the EU that can be used for ripened cheeses are subdivided into three functional groups: antimicrobials, antioxidants, and antibrowning compounds. These compounds are added to the milk vat during cheesemaking, either as antimicrobials and antioxidants, or to the cheese as surface protectors against undesirable agents. The additives approved for ripened cheeses are lisozyme, sorbic acid/sorbates, nisin, natamycin, hexamethylene tetramine (HTM), nitrates/nitrites, and propionic acid/propionates [83].

Lysozyme is an enzyme present in nature and abundantly in the egg white, from which it is usually extracted for industrial use. This enzyme has gained considerable attention as a preservative in the food industry due to its natural origin. It is a bactericidal agent against Gram (+) bacteria, such as LAB and clostridia, and to a lesser extent to Gram (−) bacteria. Thus, its industrial application is limited [84,85]. This enzyme (E-1105) has been used to prevent the “late blowing” defect in hard and semi-hard cheeses because it is effective in lysing *C. tyrobutyricum* and *C. perfringens* vegetative cells [86,87]. Previously, nitrates were added to milk instead of this enzyme; however, lysozyme is now a preferred option in order to reduce the level of nitrosamines in food [86,88]. The addition of lysozyme to milk during cheesemaking of ripened cheeses is allowed in *quantum satis* in the EU [83], but lysozyme content in cheese varies between 30 and 382 mg/kg [89] or 100 and 350 mg/kg [84]. Lysozyme added to milk (20 g/1000 L) did not compromise the functionality of the most relevant LAB in Grana Padano cheese, but significant arginine degradation was observed [87]. This fact could be due to the presence of *Lactobacillus fermentum* and *Lactobacillus rhamnosus*, which may contribute to the strain’s ability to degrade arginine. Soggiu et al. [90] assayed two milk vats containing 2.33 and 3.14 log10 MPN/L of clostridia spores, separately, and 50 mg/kg of lysozyme was added to avoid butyric clostridia growth. They found that, at a low level of clostridia in cheese, lysozyme caused a decrease in *Lactobacillus* growth but no statistically significant changes for the other genera. Contrarily, at a higher level of clostridia in cheese, no significant change was observed in the *Lactobacillus* growth. In these latter cheeses, a decrease in several enzymes involved in butyric fermentation was observed, in particular, acetate kinase and butyryl-CoA dehydrogenase. However, none of the above-mentioned studies reported information on the sensory properties of the cheeses. In this sense, a negative lysozyme effect on cheese flavor was observed when comparing pressure-treated cheeses with non-treated cheeses, although there were no significant changes in the microbiological and physicochemical properties of the cheeses [23]. One of the main problems in the use of lysozyme is its allergenic activity; thus, lysozyme addition should be declared on the labelling to satisfy EU regulations [91].

Sorbic acid is a natural compound from fruits that is often synthetically manufactured for commercial use in foods. It is added to food products such as cheese and wine as an antimicrobial preservative, due to its ability to inhibit yeast and mold growth [92]. Sorbic acid and sorbates (E-200/203) are approved in ripened cheeses by EU regulations, but only in prepacked or sliced and layered cheese at a maximum level of 1000 mg/kg (applicable to the sum of both chemical forms where the level is expressed as free acid content) or in *quantum satis* as a surface treatment [83]. Sorbic salts, such as potassium sorbate (E-202), is often used as food preservative because of its high stability, water solubility, and ease of handling [92]. The sorbate concentration necessary to inhibit microbial growth in cheese surface is around 300 mg/dm^2^, but knowledge regarding the taxonomy of the spoiling microbiota in each cheese variety is required to evaluate its effectiveness [93,94]. As their potential toxicity is low, these chemical additives are classified as Generally Recognized as Safe (GRAS) compounds, because they are rapidly metabolized by biochemical pathways similar to those for fatty acids [95]. One of the main problems regarding the use of sorbates is the change in the flavor of cheeses [93].

Bacteriocins are peptides synthesized by bacteria that inhibit or destroy other microorganisms [96]. Due to their nature, bacteriocins are rapidly digested by proteases in the human digestive tract and, although they possess antibiotic properties, they differ from antibiotics in their chemical composition and specificity action against strains of the same or related species. Nisin is a bacteriocin produced by strains of *L. lactis* that has received particular attention from the food industry due to its inhibitory effect against numerous Gram (+) bacteria, and lack of effect on Gram (−) bacteria, yeast, or mold growth [97]. This bacteriocin can permeate cell membranes through a highly efficient, pore-formation mechanism, leading to bacterial death [98]. Initially, this bacteriocin was applied to dairy products to prevent spoilage by clostridia responsible for the late-blowing defect in cheese. It is the only bacteriocin approved as a food preservative by international regulatory agencies, such as the World Health Organization (WHO), and the Food and Drug Administration (FDA) in USA [99]. Nisin (E- 234) is the only bacteriocin also approved in the EU in cheese (12.5 mg/kg ripened cheese) [83], but may be present naturally in certain ripening cheeses due to fermentation processes. The antimicrobial activity of nisin-producing strains of *L. lactis* has been demonstrated against different microorganisms, such as *Listeria monocytogenes* in ripened sheep and cow milk cheeses [100,101], and *S. aureus*, after addition to pasteurized milk [102]. Cheeses made with 0.05% (*w*/*v*) nisin added to milk were compared with control cheeses made with milk with no added nisin. After four ripening weeks, no significant differences were detected in physico-chemical parameters (moisture, pH, and tritable acidity), textural parameters, and LAB counts between both types of cheeses. In addition, significantly higher scores of flavor, texture, and overall acceptance were recorded in cheeses made with nisin added to milk [23]. At present, food safety and nutritional quality can be improved by combining the advantages of nisin addition and the application of non-thermal preservation techniques. In this regard, HHP treatments have been applied together with bacteriocin addition to achieve two main purposes, namely, the improvement of cheese microbiological safety and the acceleration of cheese ripening [51,103].

Natamycin, also called pimaricin, is an antifungal food additive (polyethylene antibiotic) produced by *Streptococcus natalensis* and other similar species [43]. Natamycin (E-235) is authorized by EU regulation only to be used as a surface treatment in hard, semi-hard, and semi-soft cheeses, at a maximum level of 1 mg/dm^2^ surface (must not be detected at a depth of 5 mm) [83]. This preservative is highly effective against mold and yeast growth, but not against bacteria, and is employed in the range of 1–20 ppm [94]. Yangilar et al. [104] assessed the efficiency of natamycin against mold growth in Kashar cheese after 60 and 90 ripening days using a 0.07% (*w*/*w*) additive concentration. No sensory differences were observed between control and natamycin-added cheeses.

The EU regulation also describes HMT and nitrates as effective additives against *Clostridium* growth. HMT has been described as a preservative (E-239) that has been only approved in Provolone cheese, with a maximum residual amount of 25 mg/kg, expressed as formaldehyde [83], in order to avoid the “late blowing” defect. Elsewhere, its use is not currently approved in USA, Australia, Korea, Japan, Australia, or New Zealand [105]. By comparison, nitrites and nitrates can be present in dairy products from endogenous and exogenous sources [106]. Their use is authorized by EU regulation (E-251/252) only in hard, semi-hard, and semi-soft cheeses with a maximum addition level of 150 mg/L in milk, or an equivalent level if added after whey removal and water addition [83]. The Codex General Standard for Food Additives authorizes a maximum level of 50 mg/kg nitrate residue in cheese [107], whereas in USA nitrates and nitrites are not approved as food additives in cheese [106]. Nitrates are effective in inhibiting *Clostridium* growth, but their reduction to nitrites can lead to risks to human health. When nitrate and nitrite maximum authorized levels are surpassed, these compounds can produce severe gastroenteritis with abdominal pain, blood in feces and urine, weakness, and unconsciousness [95,108]. At present, there is a marked tendency to replace nitrates with other preservation options in order to reduce the levels of nitrosamines in food products, which may be carcinogenic [86].

Finally, the use of propionic acid and propionates (E-280/283) is approved by EU regulation to be added in *quantum satis* only as a surface treatment in cheese [83]. These compounds were re-evaluated for their safety as food preservatives by the EFSA Panel on Food Additives and Nutrient Sources added to Food in 2014. The Panel concluded that, for foods as currently consumed, the authorized levels were correct for ripened cheeses [109]. These additives can be added to cheese during ripening and storage as antifungal and antibacterial agents to avoid problems in ripened cheeses [95]. Moreover, propionic acid can be naturally present in many semi-hard cheeses, mainly in those in which propionic acid fermentation occurs. In this respect, a study was conducted [95] of the amount of propionic acid generated during ripening and storage of 16 types of Korean hard and semi-hard cheeses, and of 40 kinds of imported cheese. The results indicated that propionic acid was not detected in domestic and imported hard cheeses, but was detected in imported semi-hard cheeses (Emmental from three European countries—France, Germany, and Switzerland—and Chevrette from Netherlands), and an average content of 18.78 mg/kg was found. The highest concentration was found in smoked cheeses made in the Netherlands (182.28 mg/kg); this high concentration may be due to the employment of *Propionibacterium* bacteria for cheesemaking. Propionic acid bacteria convert the lactic acid produced by LAB to other organic acids during ripening, resulting in an increase in the cheese pH from 5.3 to 5.8. In the case of Dutch-type cheeses made from pasteurized milk, a low pH and the presence of short-chain organic acids (lactic, acetic, citric, and propionic acids) are important factors contributing to the inhibition of *L. monocytogenes* growth [110]. Therefore, a prediction of growth/non-growth of *L. monocytogenes* in cheese containing organic acids was studied. The average minimal inhibitory concentration of undissociated propionic acid against the growth of six *L. monocytogenes* strains, tested at a pH range of 5.2–5.6 and temperature of 12 °C, was 11.0 mM. Consequently, propionic acid can play an important role in inhibiting the growth of pathogens during cheese ripening [110]. From a human health perspective, propionic acid decreases the fatty acid content in the liver and plasma, diminishes food intake, exhibits immunosuppressive actions, and enhances tissue sensitivity to insulin [111].

The additives authorized in the EU for ripened cheeses are mainly used for inhibition of Gram (+) bacteria, such as *Listeria* or *Clostridium*, and/or as antifungals. Nitrites and lysozyme exhibit negative effects on human health, the former due to the transformation into nitrosamines and the latter because of allergies that can occur in sensitive individuals. HMT is only allowed for Provolone cheese. Bacteriocins such as nisin are presently receiving increased attention because they can be used as starter or adjunct cultures with a dual purpose, contributing to flavor and food safety. Consequently, they provide fermentation and conservation simultaneously [99]. The WHO/FAO Committee approved a daily intake up to 33,000 nisin units/70 kg body weight, but the maximum nisin daily intake varies between countries [97]. From a health perspective, bacteriocins can act as modulators of human microbiome with the possibility of addressing metabolic disorders such as diabetes and inflammatory bowel disease. There is a negative aspect at the industrial scale because the use of bacteriocins has a high cost. However, this can be diminished by optimizing fermentation processes and bioengineering strains to maximize bacteriocin production [99].

Fungal spoilage is very common in cheeses, causing significant economic losses for the dairy industry. Antifungals are employed as surface treatments, but problems may occur related to cheese internal contamination with toxins and associated health hazards. Natamycin is very poorly absorbed due to its low solubility in water (approximately 40 mg/kg) [92]; therefore, there is an adequate safety margin in its applications and currently there is no concern regarding antimicrobial resistance induction [43]. Natamycin may be a good option due to its excellent efficacy compared to other fungicides, such as potassium sorbate or propionic acid, in addition to its extremely low migration from the surface to the interior of the cheese matrix [93]. Chemical preservatives may be effective and cost-effective antifungals, but they are counter to the claims of “traditional” products and concerns about health risks. Other new methods currently under study may be of interest, such as biopreservation of fermented dairy products with LAB, propionibacteria, and molds, which are able to produce a vast range of antifungal metabolites.

### 4.2. Addition of Antioxidant and Antimicrobial Plant-Based Substances

Increased consumer demand for natural foods and longer product shelf-life has led to the investigation of alternatives to replace synthetic preservatives with safe natural products [112]. Since ancient times, plants have traditionally been used as natural flavorings and preservatives, and in cheese have often been included in the form of dried plants, extracts, aqueous solutions, or essential oils (EOs). They can be applied during cheesemaking as ingredients or on the cheese surface to prevent mold growth. In this regard, significant attention has been directed to plant-based products as sources of antioxidants and antimicrobials for dairy products [113], although bioavailability, health benefits, toxicity at high concentrations, and sensory aspects must be taken into account.

Pomegranate rind (*Punica granatum*) presents antioxidant activity (AA) due to the presence of phenolic compounds that reduce lipid oxidation. Low-fat Kalari cheeses were dipped for 30 s in aqueous solutions of rind pomegranate extracts with concentrations of 1 and 2% (*w*/*w*). Cheeses were drained and stored at refrigeration temperatures (1–4 °C) for 28 days in polyethylene bags. For both concentrations of pomegranate rind extract, a significant decrease in oxidative lipid deterioration (thiobarbituric acid and FFA content), and significantly lower microorganism growth, was observed in the dipped cheeses compared to the control cheese (no extract added). Texture, flavor, and overall palatability scores were significantly higher for cheeses dipped with pomegranate rind extracts in comparison with control samples [114]. Pine needles (*Cedrus deodara* (Roxb.) Loud.) contain bioactive phytochemical compounds, phenolic compounds, and acids with AA, and antimicrobial properties. These extracts were used in the same Kalari cheese model. Aqueous concentrations of 2.5 and 5% of pine needle dry powder were applied by immersion to freshly made cheeses. For both concentrations, a significant decrease in oxidative lipid deterioration and microorganism growth was detected in the cheeses dipped with pine needle extracts. Appearance, texture, flavor, and overall acceptability scores were higher in dipped than in control cheeses [113].

Catechins are a group of flavonoid antioxidants found in many plants and fruits with AA, but these compounds become unstable after a long storage period. Three batches of cheeses made from pasteurized skimmed milk (0.1% fat) containing 125, 250, and 500 ppm of catechins were evaluated for AA. Catechins were added dissolved in 10% polyethylene glycol (PEG) (*w*/*v*). Two control batches were made: with no addition of catechin and PEG to milk, and without catechin but with PEG added to milk. Total phenolic content in cheeses increased during the ripening period, mainly in those made with the higher content of catechin. The AA values generally increased with ripening time in both control and treated batches, but the cheeses made with catechin showed higher AA than the control cheeses, suggesting that catechin remained in the treated cheeses during storage [115]. Epigallocatechin-3-gallate (EGCG) has been reported as the main catechin found in the green tea plant (*Camellia sinensis*) with important preservative potential because it inhibits foodborne diseases and contains antioxidants [112]. This compound is used at low concentrations to protect foods against undesirable off-flavor generation. EGCG was applied by immersion in 0.05 and 0.1% aqueous solutions to a low-fat Kalari cheese. Similar results for antioxidant activity, microbial growth inhibition, and sensory (texture, flavor, and overall acceptability) properties were obtained as those reported for the cheeses dipped with pomegranate rind and pine needle extracts during storage [116].

Dried rosemary (*Rosmarinus officinalis L.*) shows antibacterial and AA due to its high content of rosmarinic and caffeic acids, in addition to phenolic compounds and flavones. Semi-hard cheeses made with raw and pasteurized cow’s milk were coated with lard in a proportion of 3% (*w*/*w*) and dehydrated rosemary at 4% (*w*/*w*) after 15 ripening days. There were no significant differences in physicochemical parameters after 60 ripening days (fat, ash, acidity, and protein content), but only rosemary-coated cheeses showed a higher moisture content than uncoated control cheeses due to lard protection. Proteolysis activity was also higher in coated cheeses. No internal color differences were observed between control and rosemary coated cheeses, but the external color of coated cheese presented a more greenish tendency, particularly in pasteurized cheeses. The best sensory panel scores were recorded for the raw milk cheeses coated with rosemary due to a mild aroma and spicy flavor [117].

Novel Mudaffara cheeses were made with the individual addition of powdered spices with AA at different concentrations: 0.2% clove (*Syzygium aromaticum*), and 0.5% for both black cumin (*Nigella sativa*) and black pepper (*Piper nigrum*). Cheeses were stored in refrigeration (7 °C). After eight weeks, clove cheeses recorded the highest AA, followed by black cumin and black pepper cheeses, whereas cheeses with black pepper received the highest sensory acceptance score, followed by black cumin and clove cheeses [118]. Shan and Cay [119] analyzed the antibacterial capacity of five spice and fruit component extracts (cinnamon stick, oregano, clove, pomegranate rind, and grape seed) against two different concentrations (10 and 105 CFU/mL) of *S. aureus, L. monocytogenes*, and *Salmonella enterica* in Cheddar cheese stored at room temperature. From each sample of plant powder, 40 g was taken for extraction with 1000 mL of 80% ethanol. The treatments with the five plant extracts improved the cheese stability against lipid oxidation. Additionally, the treatments inhibited the growth of the three food-borne pathogens in the cheese. Clove extract showed the highest level of antibacterial and antioxidant activity.

In Mediterranean countries, sheep milk cheeses are relatively common in the diet. A common practice is the addition of native spices, such as saffron dried stigmas *(Crocus sativus* L.), that give cheeses a yellow-orange color. The color of saffron is due to its carotenoid content, mainly comprising crocetin esters, its bitter taste is due to picrocrocin, and its characteristic aroma is due mainly to safranal. The AA and antimicrobial properties of saffron cheeses have been described, but the number of different cheeses made with saffron is currently very scarce. Thus, it would be of interest to know the contents of potential health-promoting compounds that can be transferred from saffron to the cheese matrix [120,121,122]. Different concentrations of saffron were added (concentrations were not indicated) to pressed pasteurized sheep milk cheeses ripened for 180 days. Total mesophiles and LAB counts were lower in saffron than in control cheeses with no saffron added, suggesting that saffron may slightly slow bacterial growth. Saffron cheeses showed lower salt content and were firmer and more elastic than control cheeses. Differences in cheese color and flavor among control and saffron cheeses were observed at the beginning of the ripening period, but as ripening progressed, the differences were less evident [120,123].

Flowers are emerging as natural food additives due to their health beneficial properties. Chestnut flowers (*Castanea sativa* Mill.) and lemon balm (*Melissa officinalis* L.) may have a potential role as antioxidants and antimicrobials in foods, and in promoting consumer health. Cheeses after 1 and 6 ripening months were manually impregnated with milled dried plants and decoctions of both species. The dose applied to each cheese of chestnut flower was 799 and 248 mg of dried flower and lyophilized decoction, respectively. In the case of lemon balm, each cheese was coated with 368 and 380 mg of dried flower and lyophilized decoction, respectively. No plants were added to the control cheeses. The results showed a higher loss of moisture in cheeses with plants incorporated than in control cheeses. The addition of both dry plants appeared to be a better option than decoctions because dried plants caused greater moisture loss, smaller calcium and sodium losses, better conservation of the more abundant monounsaturated fatty acids (MUFAs) (C18:1), and a higher amount of polyunsaturated fatty acids (PUFA)s. The authors indicated that dried plants contribute to better cheese preservation compared to decoction treatments, but the cheese appearance was affected. Decoctions were slightly lower compared to preservatives, but could be a good option because they did not change the cheese appearance [124]. Similarly, they investigated the addition to cheese of basil (*Ocimum basilicum* L.), both as dehydrated leaves (1.37 g) and as decoctions (352.5 mg). After six months of storage, cheeses with basil lost more water, especially those treated with decoctions. A better maintenance of proteins in treated cheeses was observed, which may be related to the increased antimicrobial activity caused by basil components inhibiting the growth of proteolytic bacteria. The color did not change in the cheeses treated with basil decoctions, but those treated with dried leaves showed a greener appearance. An increase in the AA and a preservation of the unsaturated fatty acid content in treated cheese were observed. The authors indicated a greater functional and preservative effect of the decoction compared to the dehydrated form [125].

Another possibility to include natural preservatives is the incorporation of EOs during the cheesemaking process. Food composition can have a negative impact on the efficacy of these natural additives, particularly for protein, fat, and carbohydrate content. The EOs can reduce the growth of undesirable bacteria and molds, but it should be noted that these substances may reduce LAB growth and cause negative effects on the structure and development of cheese. Thus, their action against Gram (+) bacteria is more effective than for Gram (−) bacteria, and carbohydrates reduce the activity of EOs in some food matrices [123]. The in vitro evaluation of the antifungal activity of eight EOs (cinnamon leaf or bark, basil, ginger, lemon, peppermint, pine needle, and spearmint) indicated that the highest antifungal activity was found for cinnamon leaf and barks [126]. The main components of both cinnamon EOs were eugenol, cinnamaldehyde, and linalool. Therefore, 10 and 20 μL of cinnamon leaf and bark EOs were surface-spread for their antifungal activity during the ripening of Appenzeller cheese. The optimum concentration of cinnamon EOs (10% *v*/*v*) in cheese showed antifungal activity and did not hinder the starter culture growth. Another potential natural preservative product is oregano (*Origamun vulgare*). This plant is rich in phenolic compounds (mainly carvacrol and thymol) with antimicrobial and antioxidant activity. The antimicrobial effectiveness of different concentrations of oregano EOs (50, 100, 150, 200 mg/kg curd) was studied in pressed cheeses made with pasteurized cow’s milk. Similarly, cheeses made with oregano leaves (10 g/kg cheese) and control cheeses made without oregano were compared. After 30 ripening days at 12 °C, no negative effect on the growth and metabolism of LAB was observed in oregano cheeses. Spoilage microorganisms, such as enterobacteria, molds, and yeasts were not detected during ripening in EO cheeses, compared to control cheeses and cheeses with oregano leaves. The sensory characteristics (flavor and texture) of cheeses made with 200 µg/g of oregano EO were similar to those of cheese samples made with oregano leaves, whereas the control cheese obtained lower sensory scores [127]. Recently, the application of microencapsulated oregano EO to the cheese surface by spray drying was found to be effective to inhibit mold and yeast growth in Parmesan grated cheese during storage [128]. Rosemary EO (*Salvia rosmarinus*) was added as an antimicrobial agent in the production of sheep cheese at a final concentration of 0.2 g/kg cheese (215 mg/L milk). The cheeses made with rosemary and control batches made without rosemary were compared after five ripening months. Rosemary EO showed an antimicrobial effect in cheeses with the total inhibition of *Clostridium* spp. growth without altering LAB growth, but this EO was not effective in inhibiting mold and yeast proliferation [129].

Although “synthetic” additives in the permitted dosage are safe compounds for human health and are widely used in the food industry, there is a negative consumer perception towards them. This has led to a growing interest in more natural alternatives, such as herbs, spices, and different parts of plants that are natural and GRAS products. Such products are currently in demand by consumers [130]. Concentrations of natural compounds in food should be between 0.05 and 0.1% (by weight) (500–1000 ppm) to be effective and to ensure that sensory food quality remains unchanged [131]. Numerous studies have reported that a higher concentration of the natural compounds present in plants was needed to achieve the same antimicrobial or AA in a food matrix, such as cheese, than when applied in vitro. Natural compounds may be lost during cheesemaking due to their solubility in whey, the cheese pH, or their sensitivity to light, temperature, and oxygen [132,133]. Given their natural origin, is difficult to maintain the repeatability of their chemical composition. Therefore, because important sensory changes can occur, they have been considered as new types of cheese, or as nutritional improvements or nutraceuticals. A solution may be their inclusion as EOs in milk or during cheesemaking, or application on the cheese surface as antifungals. The latter case may address the problem often experienced by pressed cheeses with longer ripening periods. However, this has not always been successfully achieved, because the use of EOs is limited due to their impact on the sensory attributes of the cheese, especially at high concentrations that may change cheese flavor and odor [134]. Therefore, the use of plant extracts and EOs presents the difficulty of defining the specific quantity in which they should be incorporated in the cheese, in order to guarantee the expected antimicrobial and antioxidant effects without a relevant change in the sensory properties of cheese [130]. The literature presents more studies in which these compounds have been applied in fresh or short-ripened cheeses, than in longer ripened cheeses, such as hard and semi-hard cheeses.

Microencapsulation may be an up-coming technology used to ensure better stability of these interesting functional and technological compounds during cheesemaking [133], preserving their AA and antimicrobial activity throughout the shelf-life of the products. The use of microencapsulation in combination with other technological approaches, such as pulsed light, high pressure, magnetic fields, or nano-emulsions, may minimize the organoleptic impact of the plant-based substances on cheese sensory properties. Another possible solution may be their incorporation as part of edible biofilms through microencapsulation systems, active modified atmosphere packaging, and other storage materials, to improve their bioavailability in cheese without the need of a high quantity of extracts. Numerous other factors must also be assessed, such as economic costs, legislation, practical effectiveness, and organoleptic impact [130].

## 5. Packaging

Packaging is an important step in the food manufacturing and commercialization process. The objective of packaging is not only to contain food, but also to protect and maintain the quality and safety during the food’s shelf-life, at a limited business cost [135]. Cheese packaging is mainly directed to avoid certain degradation processes, such as oxidation or dehydration, protect against the growth of undesirable microorganisms and external contamination, and reduce or allow the continuance of the metabolic activities of ripening strains [136]. In packaging techniques, material properties, such as water vapor and gas barrier, and the shape and size of the package, are crucial to ensure cheese quality and safety [137]. For optimal packaging selection, it must be considered that cheese is a complex dynamic biological matrix in which several microbial, physical, and biochemical changes occur during storage. The growing consumer demand for portioned cheese sold as blocks, slices, or grated has led to the design of specific packaging conditions that ensure the desired shelf-life of this food product [138].

### 5.1. Vacuum Packaging

Vacuum packaging is the simplest and most common method to modify the internal atmosphere of a food package. Essentially, it consists of removing the air around the food, using a packaging material with low permeability to oxygen and other gases. In this situation, the packaging material collapses around the product, because the internal pressure is much lower than the atmospheric pressure; thus, the amount of oxygen inside the package is less than 1% [139,140]. This type of packaging has demonstrated its ability to reduce oxidative damage and inhibit aerobic bacteria, mold, and yeast growth, thus increasing cheese shelf-life. Due to the plastic material used, vacuum packaging prevents dehydration and weight loss in cheese, and the absorption of undesirable odors from outside [141]. However, vacuum packaging is not suitable for all cheese varieties because it can lead to undesirable consistency or texture changes, with possible modifications of the structure and appearance of the cheese [135,142]. In cheeses that have high respiratory rates, non-gas permeable packaging materials are not advisable. Such materials are also not preferred for soft structure cheeses (partially ripened or soft cheeses), due to the deformations they may undergo when reducing pressure [142]. However, this approach can be of significant interest for hard and semi-hard cheeses, both for whole pieces and fractioned presentations. These cheeses are bacteria-ripened products that have a slow ripening rate due to their low water and salt content, so the use of low gas permeability packaging materials can generate a suitable anaerobic environment in which cheeses can progressively ripen once they are packaged [138]. In addition, the vacuum condition is well tolerated by the cheese matrix due to its firmness.

Different authors have demonstrated the usefulness of vacuum packaging for preservation and cheese shelf-life extension. Thus, Parmigiano Reggiano cheese is usually sold in vacuum-packed portions of 250–300 g [143]; this is also the case for Gouda cheese. Thus, the shelf-life in both cases can be maintained up to 10 weeks in refrigerated conditions [144]. Similarly, an increase in 20 days was reported for the shelf-life of Calabrian Provola vacuum-packed cheeses compared to unpackaged cheeses [137]. This methodology was found to be useful to preserve the sensory quality of smoked cheeses cut into portions and stored up to 45 days, whereas other protective atmosphere options contributed to the accumulation of smoke-derived compounds and altered lipolysis and proteolysis processes, which led to an off-flavor in the cheese [141].

Despite the advantages of vacuum packaging, negative effects on hard and semi-hard cheeses have been reported. Vacuum packaging provides certain anaerobic conditions that favor the growth of some pathogens, such as *L. monocytogenes* and *S. aureus*, particularly in aged raw milk cheeses. The combination of vacuum packaging with refrigeration storage temperatures may limit the proliferation of these pathogens. In this regard, the decrease in these undesirable bacteria counts was greater at 10 °C after 56 days than at 4 °C after 28 days storage, probably because lower storage temperatures and vacuum conditions diminished LAB activity [145]. By comparison, due to low-pressure vacuum conditions, the cheese matrix structure may collapse, thus reducing the typical eyes of some cheese varieties, such as pre-cut Provolone, with the consequent negative impact on cheese appearance [142]. In the same manner, vacuum packaging of Parmigiano Reggiano cheese cubes resulted in a peculiar yellowish color during the first storage days, which may be associated with the oil dropping phenomena, in addition to an increase in the sourness after the first storage month [146]. Hocking and Faedo [147] described defects in Cheddar cheese due to mold formation in folds and wrinkles in the plastic packaging material. Furthermore, leaks in the vacuum packaging material may occur due to the presence of crystals (e.g., calcium lactate and tyrosine crystals) on the cheese surface, especially in extra-hard and long-aged cheeses such as Grana cheeses [148]. Calcium lactate crystals have also been observed on vacuum-packaged cheeses in which the package has lost integrity. Residual whey tends to move to the surface of the cheese or to cracks and crevices inside the cheese matrix during storage, which causes an increase in lactic acid concentration in those spaces accelerating crystal formation [149].

### 5.2. Modified Atmosphere Packaging (MAP)

This technique refers to the packaging of foodstuffs by creating an atmosphere surrounding the food having a composition different from that of the air. The objective is to provide an optimal atmospheric environment that preserves the quality and sensory food properties and, at the same time, increases food shelf-life. The gaseous composition of the atmosphere depends on the food product, and this can change during storage time due to different factors, such as food breathing, biochemical reactions, and the slow diffusion of gases through the packaging material [139]. An adequate design of the internal atmosphere is essential to guarantee food conservation during storage because, once the package is closed, there will be no option to compensate for possible atmosphere changes that may occur inside.

Gas mixtures used for cheese MAP include different percentages of carbon dioxide (CO_2_), nitrogen (N_2_), and oxygen (O_2_) in residual form. These gas mixtures are usually used together with refrigeration temperatures, thus combining preservative effects [150]. Among gases, CO_2_ is the most important from a microbiological perspective because it inhibits the growth of many microorganisms, including spoilage bacteria [135]. It is highly effective against aerobic Gram (−) bacteria and mold growth, and to a lesser extent, against Gram (+) bacteria and yeast growth. A concentration between 20 and 60% of CO_2_ in the atmosphere is required for an antimicrobial effect. At specific concentrations, CO_2_ also promotes the development of LAB. Gas mixtures rich in N_2_ present the risk of anaerobic microorganism growth and, therefore, the growth of clostridia may increase in packaged cheeses [151]. Several authors have reported that this conservation technique may extend cheeses’ commercial life with minimal changes in their sensory properties [143,152].

Furthermore, when the food products contain viable microorganisms, such as raw milk cheeses or those with starters added, the atmosphere design is more complex because it is necessary to control the microorganism growth during storage. Microorganisms are essential for the organoleptic characteristics of cheese; however, rigorous control is required to prevent cheese deterioration by anaerobic microorganism proliferation, because they may generate volatile compounds that can deteriorate the flavor [153]. High concentrations of CO_2_ are used, particularly in hard and semi-hard cheeses, to suppress microbial growth [154,155]. However, particularly for some cheese varieties, it is important to carefully adjust and control the CO_2_ concentration during storage because microorganisms involved in cheese flavor development can be affected and off-flavors may occur [26,143,155,156].

The use of MAP with 100% CO_2_ has often proved to be unsuitable for long ripened cheese packaging. It was indicated that CO_2_ inhibited spoilage microorganisms while also decreasing the concentration of many flavor compounds [157]. This high CO_2_ concentration resulted in undesirable changes in the flavor and texture of sliced Samso cheese. These cheeses presented higher concentration of aldehydes and fatty acids, and lower concentrations of alcohols and esters, than cheeses packaged with a 100% N_2_ atmosphere. The same results were observed in grated Cheddar cheese packaged with a high CO_2_ concentration [156]. The authors hypothesized that a smaller conversion rate of FFA in volatile compounds occurred in packaged cheeses due to changes in microbial metabolism. Favati et al. [142] indicated that a 100% CO_2_ atmosphere guaranteed the best microbiological stabilization of Provolone cheese wedges, although the product shelf-life, with reference to a limiting concentration of FFA, was found to be shorter than for cheese wedges packaged with lower CO_2_ concentrations. The ripening process was faster at 4 °C than at 8 °C, contrarily to that observed for lower CO_2_ concentrations. The samples stored at 4 °C showed higher acidity, and FFA and FAA content. In this regard, the CO_2_ dissolution in the cheese matrix occurring at low temperatures may contribute to the increase in acidity. Romani et al. [146] reported that Parmigiano Reggiano cheeses packed in a high CO_2_ concentration atmosphere showed flavor profiles that were distant from freshly cut, unpacked cheeses. In the same manner, sensory analysis showed a pronounced bitter taste and gradual discoloration after five storage weeks in grated Graviera cheese packaged with 100% CO_2_ and exposed to light. Color changes were related to photo-oxidation reactions [158]. Discoloration in grated Cheddar cheese packaged with a pure CO_2_ atmosphere and stored under fluorescent light was also observed [156], so that a high CO_2_ concentration MAP can produce a similar cheese appearance to that of vacuum packaging.

This is due to the high gas solubilization in cheese water and fat when kept at refrigeration temperatures, leading to a packaging collapse [139]. The balancing process between headspace atmosphere and cheese generally occurs relatively quickly within the first days after packaging [159]. Another problem related to high CO_2_ concentration in the atmosphere is the development of calcium lactate crystals in cheese, which causes cheese texture defects, and the possibility of leaks in the packaging material [160,161]. It was hypothesized that free ionic calcium combines with lactate through a mechanism involving carbonic acid and, because CO_2_ is absorbed by the cheese matrix, the pH of the whey phase is reduced and calcium concentration in whey is increased, resulting in calcium lactate crystals [162].

Results reported in the literature on the application of an atmosphere composed only of N_2_ are diverse. N_2_ acts as an inert filler gas, avoiding package collapse, and does not have detrimental effects on cheese composition and flavor [26,155,163]. Good sensory results and shelf-life extension of grated Graviera cheese packaged in a 100% N_2_ atmosphere were found [164], even under fluorescent light exposure [158]. Similarly, it was shown that semi-hard goat cheeses packaged in 100% N_2_ and in a 20% CO_2_/80% N_2_ gas mixture maintained their properties during six months of storage [165]. However, in grated Cheddar cheese packaged in an N_2_ atmosphere, changes in the volatile profile were observed, with a high concentration of methyl ketones, indicating that this cheese was highly susceptible to mold growth [156]. By comparison, in sliced Samso cheese packaged in 80 and 100% N_2_ atmospheres, the sensory panel described its flavor as buttery due to the high concentration of diacetyl and the presence of hexanal found in the sliced cheese. In this regard, it was not expected that hexanal, at low concentration, could contribute to the overall cheese flavor without providing a detectable grassy note [26]. High concentrations of N_2_ (80–100%) atmosphere did not appear to be useful to preserve the characteristics and sensory attributes of Arzúa-Ulloa cheese during storage [166]. The main disadvantage of N_2_-rich gaseous environments is the risk of the growth of anaerobic microorganisms [151].

Mixtures of CO_2_ and N_2_ are common atmospheres used in hard and semi-hard cheese packaging, but the results depend on the cheese variety [26,158]. In these conditions, it is possible to slow respiration, delay enzymatic changes, and reduce microbial growth, and thus extend cheese shelf-life [158]. High concentrations of N_2_ in the gas mixture (e.g., 30% CO_2_/70% N_2_) provided the best preservability for portioned Provolone cheese. However, a 30% CO_2_ concentration in the gas mixture provided more satisfactory results than a 10–20% CO_2_ concentration [142]. These CO_2_/N_2_ gas mixtures were able to slow proteolytic and lipolytic reactions that occur during cheese ripening more than other gas mixtures, and extend the shelf-life of cheese wedges up to 280 days. In whole Calabrian Provola cheese, a 30% CO_2_ concentration in the gas mixture also showed a positive effect on cheese quality. A decrease in total microbial count, peroxide value, and hardness score was observed, and, in consequence, the shelf-life of packed cheeses was longer than that of unpacked cheeses [137]. In Parmigiano Reggiano cheese packaged in atmospheres between 30 and 50% CO_2_, hardness and sour and sharp flavors increased after two storage months, possibly related to an early proteolysis process, followed by a reduction in both flavor attributes observed from two months to the end of the storage period [143]. These authors also reported an increase in the sharp taste during storage in Parmigiano Reggiano cheese mixtures with a low proportion of CO_2_ and a high proportion of N_2_, which was related to microbial growth and the loss of the CO_2_ antimicrobial effect [146]. The texture and flavor of smoked wedges of San Simón da Costa cheeses packaged in 20–50% CO_2_ concentration atmospheres and stored simulating retail conditions were adversely affected. An increase in sourness, piquancy, and an intense smoky odor was detected due to the high amounts of phenol, cyclopentanol, and 2-methyl-2-cyclopenten-1-one, which are considered to be compounds that negatively impact the flavor of this cheese variety [141].

O_2_ in atmospheres is usually avoided for cheese preservation because this gas promotes the growth of mold and lipid oxidation, giving rise to unpleasant flavors [167]. However, pre-cut aged white cheese packaged under reduced O_2_ (0–10%) and elevated CO_2_ (up to 75%) concentration atmospheres has been found to be a good alternative to air packaging for a minimum storage period of three months. These MAP conditions decreased lipolysis and protected the initial quality of the pre-cut aged white cheeses [168].

Active systems for atmosphere modification are being successfully used in different foods. Nevertheless, there are few references available in the scientific literature applied to cheese [167]. Floros et al. [169] proposed the use of an O_2_ absorber to control mold growth in some cheeses and thus extend their shelf-life. The use of a CO_2_ absorber caused a significant reduction in the concentration of this gas produced by microorganisms or starter cultures present in the packaged cheese. Mexis et al. [164] studied the combined use of an O_2_ absorber and an ethanol emitter to increase the shelf-life of grated Graviera cheese. Within 10 storage weeks, O_2_ and CO_2_ concentrations in the package remained below 0.01 and 1%, respectively, which was indicative of the efficiency of the system to reduce microorganism growth. Under these conditions, the shelf-life tripled with respect to the aerobically packaged cheese.

Published data show the usefulness of different packaging systems in prolonging the shelf-life of cheeses and, specifically, in preventing some of the problems that can occur in cheese after fractionation into pieces of different shape and weight. MAP packaging not only prolongs the life of cheese, but also can exert a positive effect on cheese flavor and appearance, in comparison to vacuum packaging, where the product can undergo changes in cheese texture. Vacuum packaging appears to be the most appropriate preservation technique from the sensory quality perspective because it promotes the retention of volatile compounds responsible for flavor. By comparison, MAP packaging may be particularly suitable for laminated cheeses, because it facilitates the individual separation of each piece, in contrast to the compacting effect, which can occur in vacuum packaged cheeses. Despite this, MAP packaging leads to an increase in package volume and more space is needed for storage and transport. The success of cheese packaging requires taking into account that most cheeses are highly complex ecosystems in which complicated interactions occur between starter cultures, external contaminants, storage conditions, packaging material, and the characteristics of the cheese itself. Although the potential of MAP for cheese preservation has been clearly demonstrated, specific studies must be carried out to make the appropriate selection of the gas mixture in the atmosphere and of the polymeric packaging material for each cheese variety [43,135].

Novel packaging systems incorporate new technologies, such as active packaging. This option, although more costly, may be of interest in extending cheese shelf-life and improving cheese quality and safety. As with other preservation techniques, the material used to preserve internal atmospheres is plastic, so consideration should be given to barrier effectiveness and proper sealing when applying new recyclable and biodegradable plastics to packaging.

### 5.3. Edible Coatings and Films

Edible coatings and films are thin layers based on edible biopolymers that are used to protect and promote food preservation. Traditionally, plastics and waxes have been mainly employed. However, in recent years there has been a growing trend in the use of edible and biodegradable materials for food coating. Edible coatings are applied directly in liquid form on the food surface where, after drying, a thin layer is formed. In contrast, films are dried separately, forming a stand-alone material that is then used to cover the food surface [170]. Both coatings and films have only been used recently to prolong the shelf-life of different types of cheese. The mechanisms by which edible materials increase cheese shelf-life are based on their barrier properties against moisture and gas transfer, light exposure, external contamination, and losses of volatile compounds and flavors [170,171,172].

Compounds used in the formulations of coatings and films include proteins (casein, whey protein, wheat gluten), polysaccharides (starch, cellulose, alginate, chitosan), and lipids (sunflower oil, waxes), or combinations of compounds of different chemical nature [170,173,174,175]. Proteins provide mechanical stability, and are effective barriers against gases, but their barrier effect against humidity is low [176]. Polysaccharides show a selective permeability to CO_2_ and O_2_, and because they are hydrophilic compounds, water vapor transmission through them is low [177]. Lipids are excellent barriers against moisture migration, although coatings are thicker and more fragile due to their hydrophobic behavior in comparison to protein and polysaccharide formulations [178]. Therefore, lipids mixed with proteins and polysaccharides produce coatings with better mechanical resistance and barrier properties than those of lipid coatings and films. Additives such as glycerin, sorbitol, glycol, and water, with a plasticizing function, can also be added to formulations to modify the physical properties or other functionality of the edible materials [171]. In addition, these materials can be used as carriers of others ingredients, such as antioxidants and antimicrobials, to extend the cheese shelf-life [170,179,180]. Formulation composition will condition the physical (thickness, optical, and mechanical properties) and chemical (wettability, permeability, and migration or release of active compounds) properties of edible coatings and films [171]. Furthermore, they present other valuable features, because they can be consumed together with the cheese avoiding the generation of waste, or, if not consumed, they are biodegradable [181].

One compound that has received considerable attention in recent years is chitosan, which is a cationic polysaccharide obtained from crustaceans or fungi. This compound is interesting due to its antimicrobial properties and its coating and film-forming capacity [170,182]. Chitosan-based materials have been tested on several hard and semi-hard cheese surfaces, aiming to decrease microbiological growth and extend the cheese shelf-life. A chitosan coating at 1% concentration (by weight) was used to inhibit the development of *L. innocua* inoculated into Emmental cheese cubes incubated at 37 °C [179]. Other studies have also used chitosan as an antimicrobial carrier. Fajardo et al. [180] reported an adequate control of mold and yeast growth in semi-hard Saloio cheese slices protected with a chitosan coating containing natamycin. Cui et al. [98] applied a chitosan coating with nisin-silica liposomes on a fresh Cheddar cheese surface and reported the antibacterial barrier effect of the coating against the growth of *L. monocytogenes* at 25 °C for 7 days and at 4 °C for 15 days. The use of an optimized chitosan solution also improved the conservation of Manchego cheese after 30 ripening days [183].

The application of whey protein-based edible materials in cheeses has been a research area of significant interest during recent years. The interest in this approach lies in the fact that whey is a sub-product generated during cheesemaking, and possesses interesting mechanical properties. Whey is mainly rich in β-lactoglobulin and α-albumin, and one additional advantage reported for whey proteins relates to their intrinsic bioactive properties, due to the presence of enzymes such as lactoperoxidase and lactoferrin [184]. Whey proteins to be used for coatings and films are commercialized as whey protein concentrate (WPC) or isolate (WPI) according to the protein concentrate (20–80% and >90%, respectively) [170]. WPI-based coatings incorporating different combinations of antimicrobial compounds were used to extend the shelf-life of Saloio semi-hard cheese [185]. The coatings decreased water loss, hardness, and color changes, and reduced microbial growth in the cheese samples during storage for up to 60 days. The effectiveness of various WPI-based coatings combining citric, lactic, and malic acids together with nisin revealed a synergistic preservative effect on cheese [186]. Other studies showed the effectiveness of these coatings in semi-hard cheeses applied on the first production day and maintained for 45 days [187,188]. By comparison, the use of WPC-films was found to be useful to extend the shelf-life of Cheddar cheese, maintaining its sensory properties [189]. The preservation of Kashar cheese samples using a WPI-based coating reduced the weight loss in cheese and the addition of 1.5% (*v*/*v*) ginger EO (*Zingiber officinale* Roscoe) to the coating formulation was effective against *E. coli* and *S. aureus* growth [190].

The usefulness of other proteins, such as caseins [191], and other polysaccharides for the preservation of different cheese varieties has also been reported. Such is the case of starch edible films in Port Salut [192] and Cheddar cheese samples [193]; galactomannan in Saloio cheese [194]; cellulose in Gorgonzola cheese [192,195]; carboxymethylcellulose in semi-hard Paipa type cheese [196]; or alginates and carrageenans in other cheese types [197].

Edible coatings/films have been tested with the addition of antimicrobial substances, such as nisin, natamycin, organic acids, pimaricin, or lysozyme, thus increasing the cheese storage period. Given the current preference of consumers for natural foods, research has focused on new edible materials containing natural active compounds, such as plant EOs with antioxidant and antimicrobial properties [170,193,198,199]. In this regard, the efficacy of 1.5% (*v*/*v*) of thyme (*Origanum vulgare*) and clove (*Syzygium species*) EOs incorporated into sorbitol-amended whey isolate-based film to preserve the quality and safety of a semi-hard Kashar cheese artificially contaminated with pathogenic bacteria was evaluated [200]. During storage, *E. coli* O157:H7, *L. monocytogenes*, and *S. aureus* counts decreased in coated cheeses. In a later study, the same authors demonstrated the antimicrobial activity against *E. coli* O157:H7 and *S. aureus* of a coating formulated with 1.5% (*v*/*v*) ginger EO [190]. Other authors evaluated the effectiveness by immersing semi-hard goat cheeses two or three times in chitosan liquid solutions containing 0.75% (*w*/*w*) of rosemary (*Salvia rosmarinus*) and oregano (*Origanum vulgare*) EOs. Both double-coatings prevented weight loss, improved microbial safety, and reduced lipolytic and proteolytic activity during cheese ripening. Sensory analysis revealed that cheeses double-coated with chitosan and EOs scored well, but in the case of three successive immersion treatments, flavor was negatively affected [201]. The effectiveness of 0.1 mL/L ginger (*Zingiber officinale*) EO incorporated in carboxymethylcellulose-based films against *Penicillium, Aspergilus, Geotricum*, *Mucor* spp., and *Fusarium* growth in a semi-hard cheese was reported [196]. In the same manner, the addition of 1% *v*/*v*) boldo (*Peumus boldus*) to blended films based on chitosan and gelatine decreased lipid oxidation in coated Prato cheese slices. The EO decreased the psychotropic microorganism growth, and low growth of coliforms in coated cheese slices was observed [202]. *Roselle calyx* extract–zinc oxide nanocomposites (3%) were found to enhance the shelf-life of chitosan-based coated Egyptian Ras cheese compared to uncoated cheese [203].

Food grade beeswax applied to fractionated cheese caused a significant reduction in mold growth after 4 four ripening months. Although proteolysis progress was rather slow, better results were obtained for the sensory properties and extended the shelf-life of coated cheese pieces compared to vacuum-packed cheese pieces used as control samples [204]. Vacuum-packed and wax-covered whole Kashar cheeses were also compared after 120 ripening days. In this experiment, no maturation delay or adverse effects on the quality of wax-covered cheeses were found, indicating wax can be used as a packaging material alternative to vacuum packaging [205].

In view of the above-mentioned results, edible coatings and films can be a good option to prolong cheese shelf-life, and may be even more promising when combined with nanotechnology, or with MAP, because this may allow new functionalities to be explored [170]. The motivation for greater interest and research activity in edible materials is due to the increased consumer demand for safe, healthy, and natural foods, and the concerns regarding the harmful environmental effects of non-biodegradable waste resulting from food packaging [206]. In addition, the value added to the waste obtained from several industries, and the simplicity of application, are aspects that are in favor of these approaches. The high compatibility of edible materials with multiple active compounds has been reported. EOs represent a promising approach for the development of bioactive edible films and coatings. One of the main challenges still facing edible coatings and films is their possible impact on the color and flavor of cheese, which can become unattractive for consumers, and the difficulty in obtaining a homogeneous coating on the cheese surface [170].

## 6. Cheese Post-Processing Technologies

The degree of microbial contamination that can occur during handling, slicing, and packaging steps greatly influences the quality of the final food products. In addition, post-processing cross-contamination of cheese can lead to both safety risks and significant compound losses due to spoilage, so additional control methods are needed to inactivate microorganism growth on cheese surface after the packaging step [207,208].

Light-emitting diode (LED) technology has recently received increased attention as a novel preservation technology for bacterial inactivation. Bacterial cells are excited when exposed to light photosensitizers, such as endogenous porphyrin, resulting in the release of reactive oxygen species, which may damage cell membranes, enzymes, proteins, or deoxyribonucleic acid (DNA), leading to cell death [209,210]. It has been recently shown that 460–470 nm LED illumination was able to inactivate *L. monocytogenes* and *Pseudomonas fluorescens* growth on the surface of packaged sliced cheese, especially when combined with refrigeration temperatures [211].

Pulsed ultraviolet (UV) light is more advantageous than continuous UV light in terms of microbial inactivation efficiency [212], and pulsed UV light can significantly reduce the microbial growth on the cheese surface [213]. Several authors have reported that the application of UV light in combination with other preservation treatments (refrigeration, MAP, antimicrobial substances) has significant benefits for safety and the shelf-life of cheese [211,214,215]. Therefore, these new technologies can represent a good option for minimizing deterioration phenomena during cheese storage.

The addition of antimicrobial substances (2.5% nisin and 50 mg/L natamycin solutions) to the cheese surface may synergistically increase the antimicrobial effectiveness of pulsed UV light (9.22 J/cm^2^), when antimicrobial substances are added after the light treatment [207]. Similarly, the combination of pulsed UV light (1.2–6 KJ/m^2^) and antimicrobial (0.001% sodium benzoate/30% citric acid) starch films were effective in reducing *L. innocua* growth on the surface of Cheddar cheese. However, significant changes in physico-chemical properties of the treated cheeses were observed after seven days of refrigerated storage [216]. These results highlight the opportunity to use pulsed UV light as a final preservative treatment in cheese pre-packaged with clear materials, and may become a very attractive solution to mitigate surface cheese contamination in manufacturing, distribution, and retail environments [213]. It has been demonstrated that pulsed UV light (44 J/cm^2^) has the potential for post-processing decontamination of the surface of semi-hard cheese [217]. Although the current trends are based on minimizing the impact on cheese quality parameters by combining treatments applied at low intensity, the combination of preservative treatments may not always result in synergistic effects, and interactions between treatments need to be studied before being applied at the commercial level [207].

Food irradiation has the ability to disrupt the microorganism DNA, thereby prolonging shelf-life and enhancing food safety, without a detrimental effect on the food sensory and nutritional quality when applied at an appropriate dose [218]. Ionizing irradiation at less than 3 kGy has proven to be an effective technology to control *L. monocytogenes* growth in cheese [219]. However, there are some discrepancies regarding the occurrence of off-flavors and depreciation of sensory quality in cheese. Probably for this reason, only some studies have been found in the scientific literature on cheese irradiation. Nevertheless, it appears that lower radiation doses do not affect the composition of different cheese types [218,220]. In Cheddar cheese, off-flavors were detected immediately after E-beam treatment, although off-flavors progressively disappeared during storage when radiation doses were lower than 2 kGy [221]. X-ray radiation at a high dose of 0.8 kGy was suitable to reduce microbial contamination of packaged sliced Cheddar cheese without affecting product quality; thus, X-ray radiation may be applied as a new post-processing antimicrobial technology for cheese preservation [208]. Ras cheese treated with γ-irradiation (5–15 kGy) showed higher degradation of biogenic amines without any detrimental changes in cheese chemical composition after six storage months compared to non-irradiated cheese samples. The results of the irradiation treatment resulted in adequate cheese suitability and wholesomeness, together with consumer acceptability of the sensory attributes [222].

In summary, cheese irradiation is found to be safe, with a potential application in the preservation and shelf-life extension in the case of certain cheeses. However, full acceptance of this preservation technology, and its incorporation into the food dairy industry, is slow and often controversial. Further studies are needed for the successful adaptation at the industrial level [218].

## 7. Conclusions

All the technologies described in this review require increased attention in relation to the presence of highly resistant microorganisms, such as spore-formers, molds, or certain anaerobic microorganisms. The potential impact of preservation technologies on biochemical and sensory characteristics of cheese should not be forgotten. Therefore, each type of cheese needs a specific preservation treatment and optimal application conditions in order to ensure cheese quality and safety during storage.

Some novel freezing processes are being improved to enhance the performance of frozen cheeses. Other innovative practices having significant potential at an industrial scale include HHP, which can be applied individually or in combination with other preservation technologies to cheese matrices. New packaging systems may be of interest, specifically to prevent some problems that can occur in fractionated cheeses. By comparison, cheese post-processing technologies remain controversial, and are little used by the dairy industry and require further studies for wider application.

At present, a major demand from food consumers in numerous developed countries is strongly related to reducing the use of non-biodegradable plastic, which is often used for food packaging and leads to the accumulation in the environment of waste materials. However, currently, plastics are commonly used as packaging materials in freezing, HHP, vacuum packaging, and MAP technologies. To minimize this problem, new recyclable and biodegradable materials (edible coatings and films), which can provide similar protection and barrier functions as plastic materials, should be thoroughly investigated. In this regard, there is also a growing consumer demand for the use of natural preservatives, mostly from plants. These compounds added as preservatives to cheese may offer health benefits when consumed, but their bioavailability and potential impact on the sensory properties of cheese should be studied.

The information discussed in this review provides evidence that preservation studies play a fundamental role in increasing the shelf-life of hard and semi-hard cheeses, and are of significant importance for the profitability of the dairy sector. Other relevant aspects to be considered are the contribution of the preservation techniques to the sustainability of the food chain and consumer preferences.

## Data Availability

No new data were created or analyzed in this study. Data sharing is not applicable to this article.
